# Fabrication and characterization of n-type Ge_1−x_Sn_x_- and Si_1–x–y_Ge_y_Sn_x_-on-SOI junctionless transistors

**DOI:** 10.1038/s41598-025-31272-y

**Published:** 2025-12-26

**Authors:** Oliver Steuer, Sayantan Ghosh, Daniel Schwarz, Michael Oehme, Sebastian Lehmann, René Hübner, Ciarán Fowley, Artur Erbe, Shengqiang Zhou, Manfred Helm, Gianaurelio Cuniberti, Slawomir Prucnal, Yordan M. Georgiev

**Affiliations:** 1 Institute of Ion Beam Physics and Materials Research, Helmholtz-Zentrum Dresden-Rossendorf, Bautzner Landstraße 400, 01328 Dresden, Germany; 2 Institute of Semiconductor Engineering, University of Stuttgart, Pfaffenwaldring 47, 70569 Stuttgart, Germany; 3 Institute for Metallic Materials, Leibniz Institute of Solid State and Materials Science, 01069 Dresden, Germany; 4 Institute of Materials Science and Max Bergmann Center, Technische Universität Dresden, 01069 Dresden, Germany; 5 Institute of Electronics, Bulgarian Academy of Sciences, 72, Tsarigradsko Chausse Blvd., 1784 Sofia, Bulgaria; 6 Center for Advancing Electronics Dresden, Technische Universität Dresden, 01062 Dresden, Germany

**Keywords:** Engineering, Materials science, Nanoscience and technology, Physics

## Abstract

Ge_1−x_Sn_x_ and Si_1−x−y_Ge_y_Sn_x_ alloys are promising materials for future nanoelectronic applications owing to their high carrier mobilities and CMOS compatibility. However, ternary Si_1−x−y_Ge_y_Sn_x_ transistors have only theoretically been discussed, and there are only a few reports on lateral n-type Ge_1−x_Sn_x_ transistors to benchmark their material performance. The low equilibrium solid solubility of Sn in Si_1−x_Ge_x_ (less than 1 at%) requires device fabrication processes at temperatures below the growth temperature of Si_1−x−y_Ge_y_Sn_x_ (x > equilibrium solubility) or at non-equilibrium conditions. Therefore, Si-based processes need to be adjusted according to the materials requirements. A relatively easy-to-fabricate device concept are junctionless field effect transistors, which operate as a gated resistor. In this work, we use Ge_0.94_Sn_0.06_ and Si_0.14_Ge_0.80_Sn_0.06_ grown on silicon-on-insulator substrates to fabricate and characterize lateral n-type Ge_1−x_Sn_x_ and Si_y_Ge_1−x−y_Sn_x_ junctionless field effect transistors. The transistors were structurally characterized by top-view scanning electron microscopy and cross-sectional transmission electron microscopy. Electrical characterizations by transfer characteristics show the first working n-type Ge_1−x_Sn_x_ and Si_1−x−y_Ge_y_Sn_x_ hetero-nanowire transistors, achieving on/off-current ratios of up to eight orders of magnitude.

**Supplementary Information:**

The online version contains supplementary material available at 10.1038/s41598-025-31272-y.

## Introduction

Germanium-tin (Ge_1−x_Sn_x_) and silicon–germanium–tin (Si_1−x−y_Ge_y_Sn_x_) alloys are promising CMOS-compatible future materials to overcome material-related limits in the silicon-based transistor technology. The incorporation of Sn into the Ge or Si_1−y_Ge_y_ lattice allows an effective band gap engineering. Hence, the Sn-containing group-IV alloys have promised superior mobilities of up to 6000 cm^2^/Vs for electrons and 4500 cm^2^/Vs for holes^1–5^ if the materials can be fabricated in high quality. Transistors based on high carrier mobility channel materials are desired to outperform conventional group-IV transistors in terms of: (i) higher drain-source currents by using the same gate potential, (ii) higher cut-off and operating frequencies, (iii) lower supply voltages, and (iv) reduced power consumption. Ge_1−x_Sn_x_ and Si_1−x−y_Ge_y_Sn_x_ alloys are typically grown in the same epitaxy reactors as Si and Si_1−y_Ge_y_ by chemical vapor deposition^6,7^ or molecular beam epitaxy (MBE)^8,9^. The vertical growth of high-quality Ge_1−x_Sn_x_ on Ge-buffered Si substrates in combination with optimized process steps recently allowed the fabrication of well-performing vertical gate-all-around n-type Ge_0.95_Sn_0.05_ field effect transistors (FETs) with small subthreshold swings (*SS*) of 76 mV dec^−1^ and *I*_*on*_/*I*_*off*_-ratios of about 7.5 × 10^3^^10^. This shows the feasibility of fabricating fast Sn-containing transistors and the existence of suitable process windows. On the other hand, most of the integrated circuits in industrial applications are still in a planar or lateral configuration and use p-n junctions or insulating substrates to isolate the devices from the substrates. The invention of insulating substrates such as silicon-on-insulator (SOI) improved the transistor performance significantly^11,12^. Unfortunately, the fabrication of Ge_1−x_Sn_x_OI is technologically challenging^11,13–15^, and Si_1−x−y_Ge_y_Sn_x_OI has not been reported yet. A cheaper approach would be the direct growth of Ge_1−x_Sn_x_ and Si_1−x−y_Ge_y_Sn_x_ on SOI substrates. However, the growth can suffer from the large lattice mismatch between the Sn-containing alloy and Si. The general feasibility of growing single-crystalline Ge_1−x_Sn_x_ directly on Si substrates has been demonstrated^16–18^, and defect densities of about ~ 10^7^ cm^−2^^18^ were estimated. Furthermore, increasing the Si concentration in Si_1−x−y_Ge_y_Sn_x_ can reduce the lattice mismatch, and post-growth pulsed laser annealing can improve the material quality, as recently shown in reference^19^. To verify the material quality and processing technology of Ge_1−x_Sn_x_ or Si_1−x−y_Ge_y_Sn_x_ on SOI, transistors can be used as suitable electrical devices. One relatively simple device concept is the junctionless field effect transistor (JLFET), which requires a uniform doping concentration in the source, channel, and drain regions. The absence of junctions simplifies the fabrication process, avoids dopant-diffusion-related issues, and allows excellent short-channel characteristics^20–22^. The operation of a JLFET is based on the depletion and accumulation of conducting charge carriers within the highly doped (~ 10^19^ cm^−3^) channel region, which is fundamentally different from the well-known inversion-mode MOSFETs^23^. An explanation of the general JLFET functionality can be found in the supplementary materials, part A, and and its related illustration in Supplementary Figure S1. Based on the channel geometry and gate configuration, many different depletion-based JLFET concepts were proposed ^24^. JLFETs based on nanowires (NW) allow efficient device control by the gate with subthreshold swings as low as 70 mV dec^−1^^22^.

In this paper, a fully CMOS-compatible top-down fabrication approach for lateral n-type Ge_1−x_Sn_x_ and Si_1−x−y_Ge_y_Sn_x_ on SOI hetero-nanowire junctionless transistors (JNT) is shown. The channel region of a JNT has the shape of a nanowire, allowing better gate control than for thin-film JLFETs. Top-view scanning electron microscopy (SEM) and cross-sectional transmission electron microscopy (TEM) are used to characterize the JNTs structurally. The electrical JNT performance is investigated by the transfer characteristics with applied potentials from the top gate (TG), back gate (BG), and a combination of both gates.

## Experimental part

### Fabrication of n-type JNTs

**Fig. 1:**
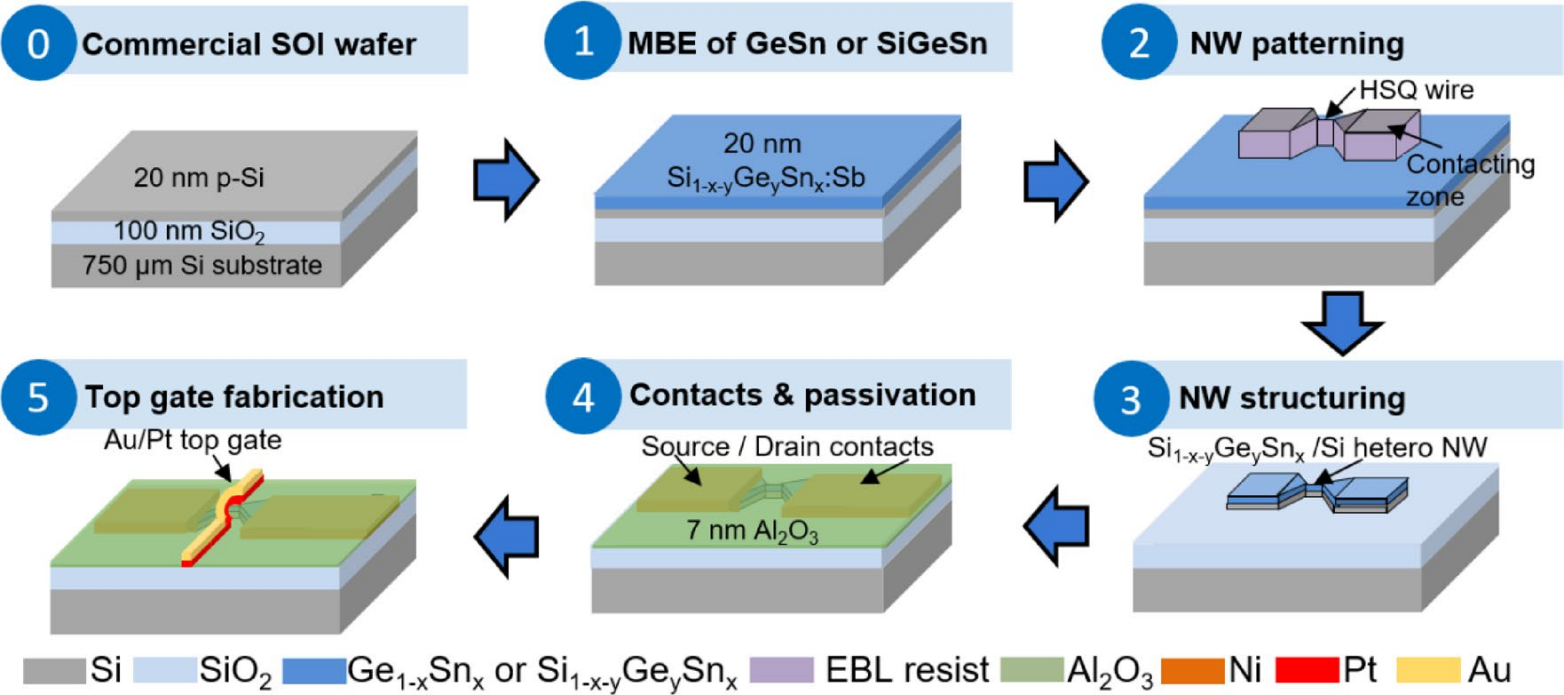
Top-down fabrication approach of lateral n-type Ge_1−x_Sn_x_ or Si_1-x-y_Ge_y_Sn_x_ on SOI hetero-NW JNTs.

**Fig. 2:**
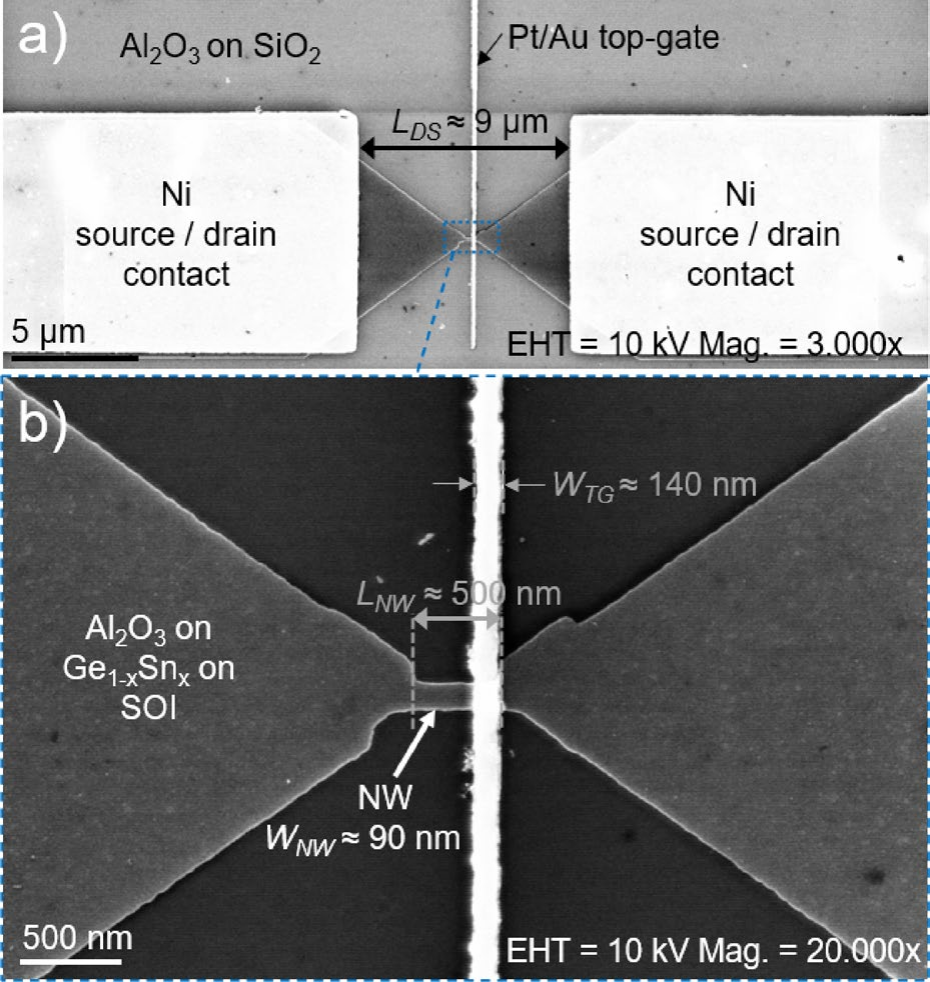
Overview (**a**) top-view SEM image of the Ge_1−x_Sn_x_ on SOI JNT after top gate fabrication and the magnified image section around the NW (**b**). The layer stack at selected positions and the distance between the source and drain contact *L*_*SD*_, NW width *W*_*NW*_, NW length *L*_*NW*_, and the top gate (TG) width *W*_*TG*_ are allocated.

The process flow for the fabrication of lateral n-type Ge_1−x_Sn_x_ and Si_1−x−y_Ge_y_Sn_x_ on SOI JNTs using a top-down gate-last approach is depicted in Fig. 1, and a processed JNT is shown in Fig. 2. A 20 nm-thick Ge_0.94_Sn_0.06_ or Si_0.14_Ge_0.80_Sn_0.06_ layer, in situ doped with antimony (Sb) in the range of 5 × 10^19^ cm^−3^, was epitaxially grown by MBE on SOI substrates having a 20 nm top Si layer (step 1). Details about the growth and a comprehensive characterization of the as-grown materials can be found in reference^19^.

Afterwards, [110]-oriented single NWs with a length *L*_*NW*_ of 0.5 µm and a width *W*_*NW*_ of 90 nm (Ge_1−x_Sn_x_) or 219 nm (Si_1−x−y_Ge_y_Sn_x_) were fabricated by GeO_x_ etching^25^, spin coating of the negative resist hydrogen silsesquioxane (HSQ), electron beam lithography (EBL), 25% tetramethyl-ammonium hydroxide (TMAH) and MF-319-based development (step 2)^26^, and inductively-coupled plasma reactive ion etching (ICP-RIE) with chlorine-based chemistry (step 3).

After stripping the HSQ resist by wet etching with 1% hydrofluoric acid (HF) deionized water (DI) solution, the NW dimensions were measured using top-view SEM imaging. The source/drain contacts were fabricated by spin coating the positive EBL resist ZEP520A^27,28^, EBL exposure, opening of the exposed windows by development, 1% HF:DI native oxide etching, thermal evaporation of 50 nm-thick Ni contacts, and lift-off. The fabricated source/drain Ni contacts have a distance of *L*_*DS*_ ≈ 9 µm and cover the Ge_1−x_Sn_x_ or Si_1−x−y_Ge_y_Sn_x_ NW contact zones, as visible in Fig. 2a). Afterwards, the low-quality native GeSnO_x_ or SiGeSnO_x_ on the NW structures were etched with the acetic acid:DI etching approach^25^, and 7 nm aluminum oxide (Al_2_O_3_) (equivalent oxide thickness EOT ≈ 3 nm) were deposited by atomic layer deposition (ALD) using Trimethylaluminium (TMA) and water (H_2_O) as precursors to passivate the NW surface (step 4). In step 5, the TG was fabricated by PMMA spin coating, opening windows for the TG with EBL exposure and structure development, followed by a deposition of 25 nm-thick Pt and 5 nm-thick Au and lift-off. Pt was selected as the TG metal because of the inherent low resistivity and high work function of 5.6–6.4 eV^29^, which allows an effective depletion of e^−^ in the n-type NW channel. Au was used as a ductile metal to ensure an entire electrical contact around the NW.

### Characterization of n-type JNTs

The fabricated JNTs were structurally analyzed by cross-sectional TEM and electrically by measuring the transfer characteristics. An image-C_s_-corrected Titan 80–300 microscope (FEI) operated at an accelerating voltage of 300 kV was used for recording high-resolution TEM images. High-angle annular dark-field scanning transmission electron microscopy (HAADF-STEM) imaging and spectrum imaging analysis based on energy-dispersive X-ray spectroscopy (EDXS) were performed at 200 kV with a Talos F200X microscope equipped with a Super-X EDX detector system (FEI). Before (S)TEM analysis, the specimen mounted in a high-visibility low-background holder was placed for 10 s into a Model 1020 Plasma Cleaner (Fischione) to remove potential contamination. TEM lamellae preparation was done by in situ lift-out using a Helios 5 CX focused ion beam (FIB) device (Thermo Fisher) along the Pt TG, along the NW, and at the Ni source/drain contact. The probe system PA200 from Süss Microtec equipped with a semiconductor characterization system SCS-4200 and a switching matrix with Model 7174A 8 × 12 low-current matrix card from Keithley Instruments was used for electrical measurements. The measurements were carried out in a grey room environment (class 100000). The temperature was kept at 25 °C with a temperature control ATT low temp system C200-60 from Advanced Temperature Test Systems GmbH (ATT). The presented transfer characteristics were performed with a double hysteresis sweep of the gate, and the presented curves belong to the second sweep. In particular, the TG was swept between − 4 V and + 4 V and the BG between − 40 V and + 40 V. For the transfer characteristics, the supply voltage *V*_*DS*_ was limited to 0.5 V, since the alloys are metastable and the SOI substrate can suffer from localized self-heating effects during device operation because of the limited heat dissipation through the underlying insulator^30,31^. Details about the extraction of the JNT figures of merit can be found in the supplementary materials, part B, and and its related illustration Supplementary Figure S2.

## Results and discussion

**Fig. 3:**
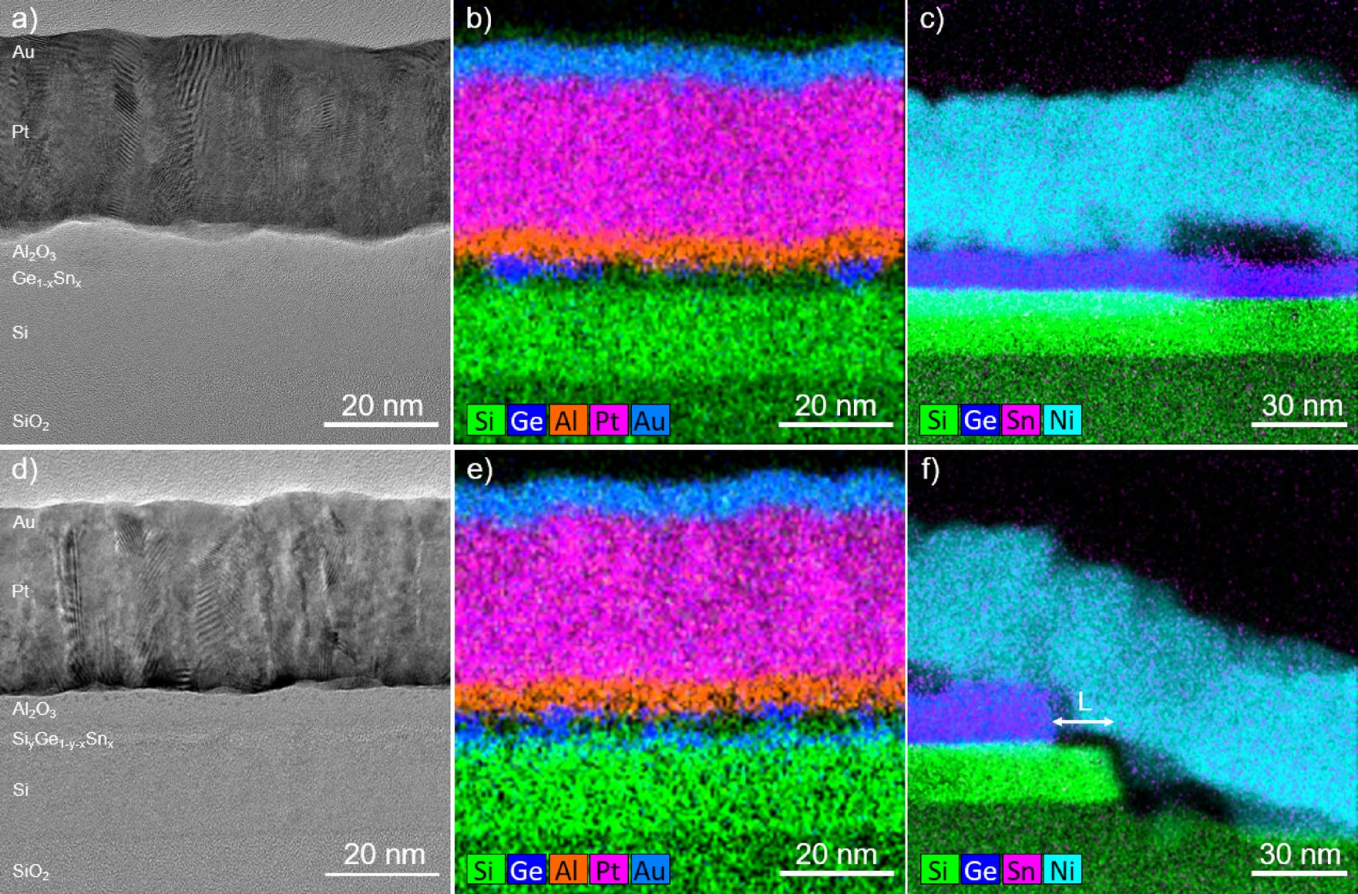
Cross-sectional TEM-based analyses of the Ge_1−x_Sn_x_ (**a**–**c**) and Si_1−x−y_Ge_y_Sn_x_ (**d**–**f**) JNTs for the center of the Pt/Au top gate (**a**, **b**, **d**, **e**), and at the Ni source/drain contact (c and f). The EDXS-based element distribution analyses in (**b** and **c**) show superimposed maps for Si (green), Ge (blue), Pt (magenta), Au (light blue), Al (orange), and Ni (cyan).

A cross-sectional TEM-based analysis of the fabricated JNTs is shown in Fig. 3. The TEM images in Fig. 3a) and d) as well as the corresponding element distribution maps in Fig. 3b) and e) taken from the TG confirm the general layer stack. However, note that the Ge_1−x_Sn_x_ layer in Fig. 3a) and b) as well as the Si_1−x−y_Ge_y_Sn_x_ layer Fig. 3d) and e) are only below 4 nm thick and do not appear fully continous along the TG cross-section. This significantly reduces the n-type conducting cross-section of the hetero-NW, but allows the depletion of the conducting cross-section more efficiently. Furthermore, detecting Sn within these ultrathin layers appears to be difficult, as discussed in Supplementary Materials, Part C, and its related illustration Supplementary Figure S3. An approximately 7 nm-thick Al_2_O_3_ layer separates the entire hetero-NW structure from the Pt/Au TG. Areas without Ge_1−x_Sn_x_ or Si_1−x−y_Ge_y_Sn_x_ have an additional native SiO_2_ layer underneath the Al_2_O_3_, since the GeO_x_ etching using acetic acid:DI solution is not capable of removing SiO_2_. The polycrystalline Pt/Au TG appears homogeneous on top of the NW. Figure 3c) and f) show the Ni contact zones: in the center of the contact area for Ge_1−x_Sn_x_ in c) and at the edge of the contact zone for Si_1−x−y_Ge_y_Sn_x_ in f). Even without any contact formation annealing, local diffusion of Ni into Ge_1−x_Sn_x_, Si_1−x−y_Ge_y_Sn_x_, and the upper part of the underlying Si is observed at the contacts; it most likely occurred during the metal deposition process. The Ni-Ge_1−x_Sn_x_ and Ni-Si_1−x−y_Ge_y_Sn_x_ contacts are between 10 and 20 nm thick. The presence of a much thicker Ge_1−x_Sn_x_ and Si_1−x−y_Ge_y_Sn_x_ layer at the contacts compared to the NW indicates that the acetic acid:DI etching is capable of etching of the defect-rich parts of the alloys. Furthermore, the horizontal distance between the top-Si of the SOI and the Si_1−x−y_Ge_y_Sn_x_, highlighted by the “L” in Fig. 3f), indicates a lateral underetching during the RIE process of about 21 nm in for Si_1−x−y_Ge_y_Sn_x_ and 18 nm for Ge_1−x_Sn_x_. A similar underetching was also observed at the NW structures after fabrication step 3 (see Fig. 1) by using top-view SEM imaging.

**Fig. 4:**
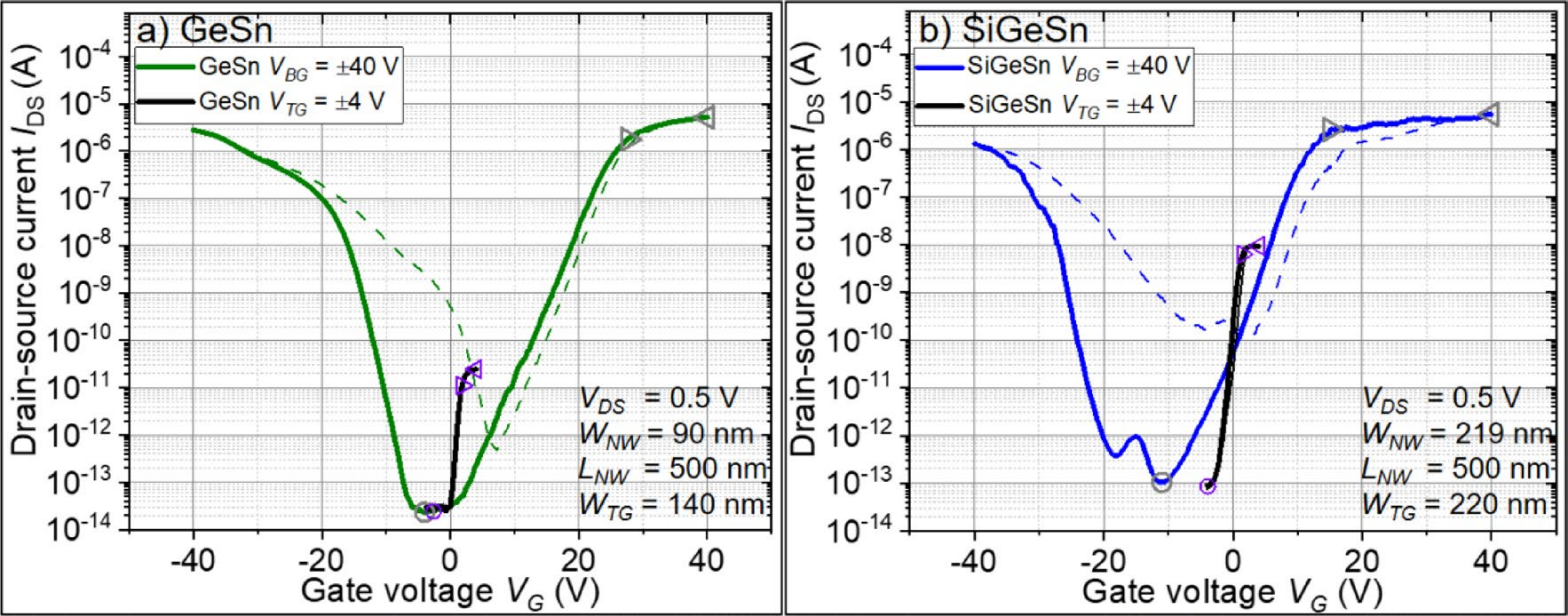
Back-gated (BG) and top-gated (TG) transfer characteristics of the fabricated n-type Ge_1−x_Sn_x_ (**a**) and n-type Si_1−x−y_Ge_y_Sn_x_ (**b**) JNT. Solid lines indicate the forward-sweep, and the backward-sweeps are presented as dashed lines. The open symbols highlight the off-current *I*_*off*_ (circle), on-current *I*_*on*_ (triangle pointing right), and maximum current in accumulation mode *I*_*max*_ (triangle pointing left). Furthermore, the grey symbols are related to the BG, and the purple symbols belong to the TG device parameters. Note: The back-gated and top-gated characteristics share the same common axis.

The back-gated transfer characteristics of the n-type Ge_1−x_Sn_x_ and Si_1−x−y_Ge_y_Sn_x_ on SOI JNTs are shown in Fig. 4. Both devices have an n-branch towards positive *V*_*BG*_ and a p-branch towards negative *V*_*BG*_. Between both branches, the devices turn off at the *I*_*DS*_ minimum (*I*_*off*_). The n-branch originates from the highly n-type-doped Ge_1−x_Sn_x_ and Si_1−x−y_Ge_y_Sn_x_ thin films. The p-branch likely originates from the contribution of the wider and thicker p-Si layer of the SOI substrate. This is in line with the presence of both branches in the transfer characteristics of p/n-stacked poly-Si junctionless FETs reported in ref.^32^. For JNTs, the on-current (*I*_*on*_) is the current in flat band condition and *I*_*max*_ the current in the accumulation mode, as explained in Supplementary materials part A) more in detail. The *I*_*on*_*/I*_*off*_*-*, *I*_*max*_*/I*_*off*_–ratio, and the subthreshold swing (*SS*) of the back-gated characteristics are 7.7 × 10^7^, 8.3 × 10^8^, and 3050 mV/dec. (Ge_1−x_Sn_x_), and 2.5 × 10^7^, 5.5 × 10^7^, and 2370 mV/dec. (Si_1−x−y_Ge_y_Sn_x_), respectively. The *SS* are relatively large due to the 100 nm-thick buried SiO_2_ and the defect containing Ge_1−x_Sn_x_ or Si_1−x−y_Ge_y_Sn_x_ layers ^19^.

Controlling the JNTs only by the TG shows clear n-type behavior and a much faster device switching. However, the on-currents (*I*_*on*_) are significantly lower than the back-gated result. The general difference between the TG and BG cases can be understood by the different active device architectures. In the back-gated case, the BG potential affects the whole JNT structure, including the source/drain contacts. This lowers the total alloy resistance and improves the Schottky-like contacts due to enhanced band bending^33^, which increases *I*_*on*_. In the case of the top-gated JNT, the gate width covers only a fraction of the NW, which allows the control of the JNT but does not allow carrier injection away from the TG.

**Fig. 5:**
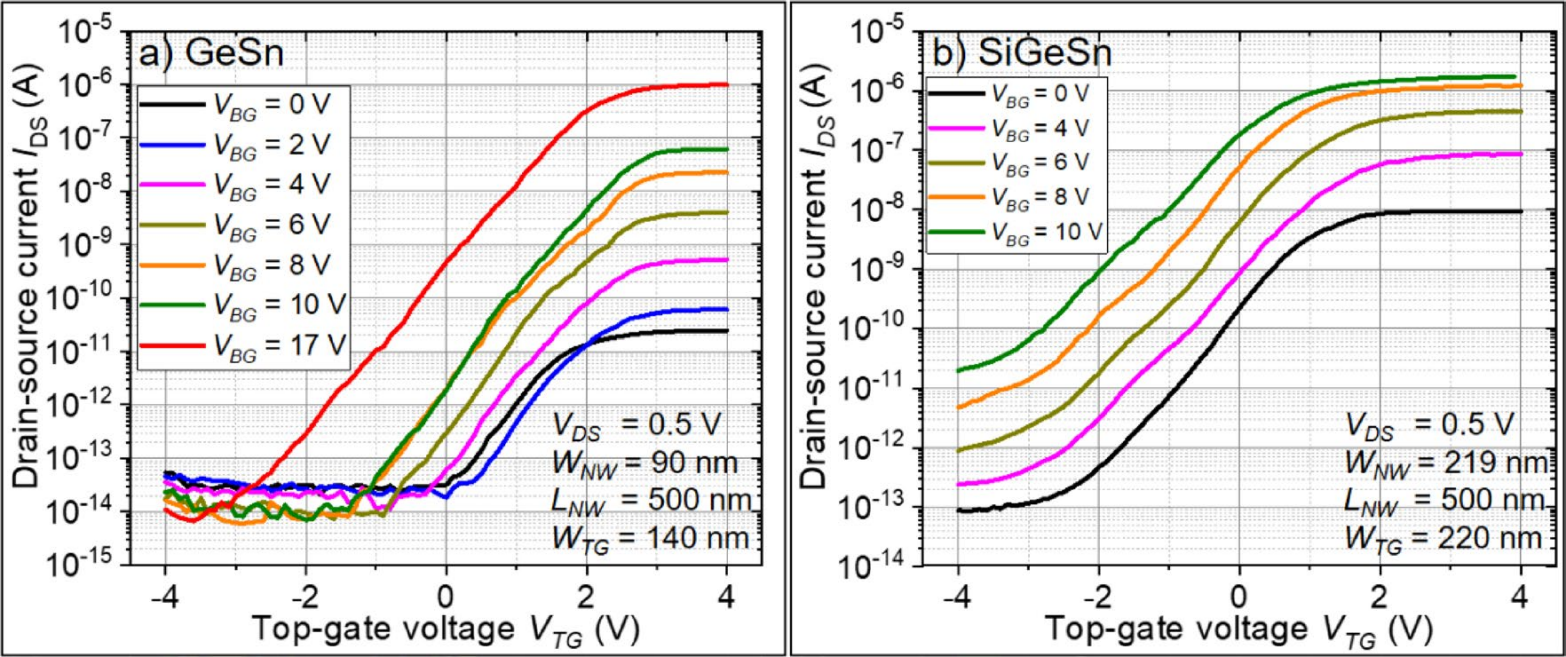
Top-gated transfer characteristics of the Ge_1−x_Sn_x_ (**a**) and Si_1−x−y_Ge_y_Sn_x_ (**b**) JNT in dependence on a constant back-gate potential *V*_*BG*_.

In order to benefit from the higher *I*_*DS*_–ratios in the back-gated case and the steeper switching in the top-gated case, a constant *V*_*BG*_ was applied and the TG was used to control the JNT, as shown in Fig. 5. This device configuration simulates a kind of gate-all-around structure and allows one to judge the entire performance of these first reported n-type Ge_1−x_Sn_x_ and Si_1−x−y_Ge_y_Sn_x_ JNTs. The *I*_*DS*_-*V*_*TG*_ characteristics of Ge_1−x_Sn_x_ in Fig. 5a) show a relatively constant *I*_*off*_ of around 10 fA, nearly independent of *V*_*BG*_. On the other hand, *I*_*on*_ and *I*_*max*_ increase significantly with increasing *V*_*BG*_, as discussed in more detail below. For Si_1−x−y_Ge_y_Sn_x_ in Fig. 5b), *I*_*on*_ and *I*_*max*_ increase with increasing *V*_*BG*_. Unfortunately, *I*_*off*_ also increases, since the much wider NW cannot be fully depleted using only the TG.

**Fig. 6:**
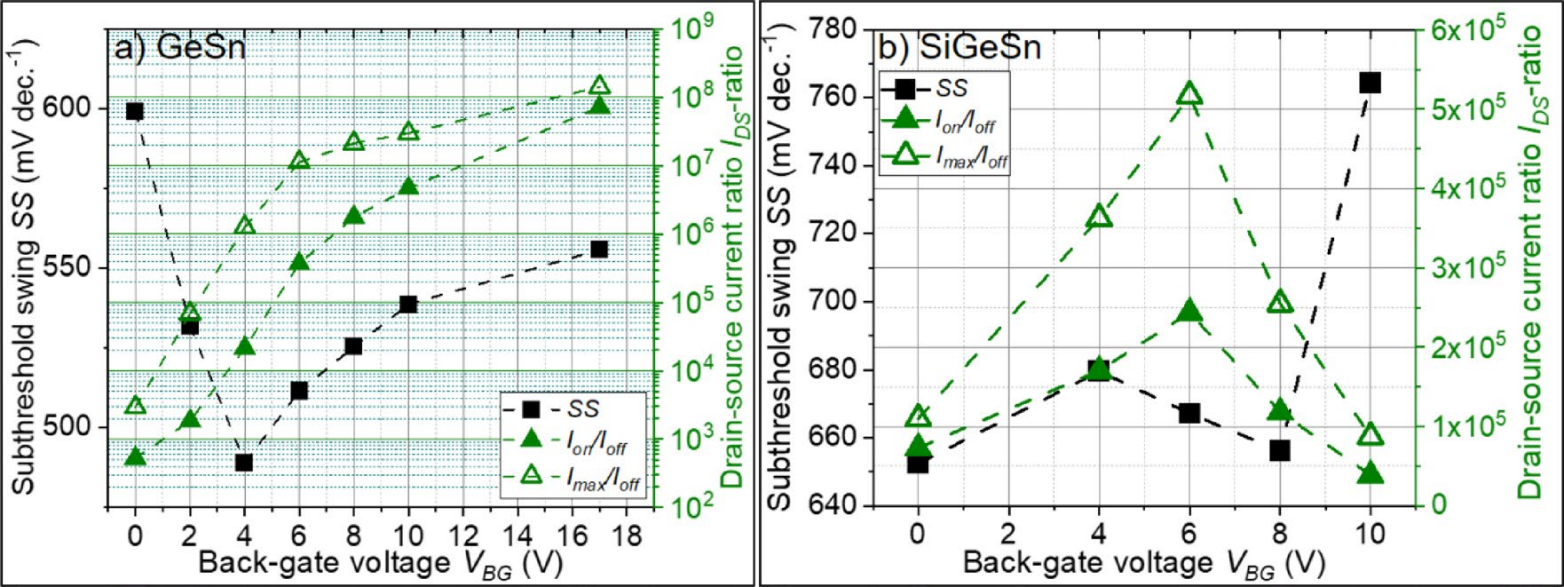
Extracted minimum subthreshold swing *SS* and drain-source current *I*_*DS*_-ratios of the JNTs shown in Fig. 5a) for Ge_1–x_Sn_x_ and b) for Si_1–x–y_Ge_y_Sn_x_ in dependence on a constant applied *V*_*BG*_.

The JNTs in Fig. 5 were analyzed in terms of their *SS*, *I*_*on*_*/I*_*off*_, and *I*_*max*_*/I*_*off*_, and the results are summarized in Fig. 6. In the case of the Ge_1−x_Sn_x_ JNT, the *SS* decreases for small *V*_*BG*_, most likely due to the gate-all-around like device structure. However, for higher BG potentials (*V*_*BG*_ > 4 V), the electrical fields of the gates seem to interact with each other. This can be the reason for the slight increase of the *SS* for *V*_*BG*_ > 4 V in Fig. 6a). On the other hand, the *I*_*DS*_-ratios increase significantly with increasing *V*_*BG*_ from *I*_*on*_*/I*_*off*_ = 5.3 × 10^2^ and *I*_*max*_*/I*_*off*_ = 2.9 × 10^3^ at V_BG_ = 0 V to *I*_*on*_*/I*_*off*_ = 7.2 × 10^7^ and *I*_*max*_*/I*_*off*_ = 1.4 × 10^8^ at *V*_*BG*_ = 17 V, which is close to the excellent *I*_*DS*_-ratios of the back-gated results. The *SS* of Si_1−x−y_Ge_y_Sn_x_ in Fig. 6b) are generally higher, since the large *W*_*NW*_ prevents steep switching by the TG, and the additional BG potential cannot improve the *SS*. This result suggests that shrinking the device dimensions will lead to significantly smaller *SS*. In the past, *SS* of 70 mV/dec. were reported for n-type Si junctionless transistors with a NW diameter of about 10 nm^22^, which is close to the physical limit. Owing to the inability to turn off the Si_1−x−y_Ge_y_Sn_x_ JNT completely, the *I*_*DS*_-ratios can be only slightly boosted by applying the additional *V*_*BG*_ from *I*_*on*_*/I*_*off*_ = 7.3 × 10^4^ and *I*_*max*_*/I*_*off*_ = 1.1 × 10^5^ at V_BG_ = 0 V to *I*_*on*_*/I*_*off*_ = 2.4 × 10^5^ and *I*_*max*_*/I*_*off*_ = 5.2 × 10^5^ at *V*_*BG*_ = 6 V. A brief benchmark of n-type Ge_1−x_Sn_x_ and Si_1−x−y_Ge_y_Sn_x_ transistors and some future prospects of these devices are summarized in Supplementary materials part D) as well as in the related illustrations Supplementary Figure S4 and Supplementary Table S1.

## Conclusion

Lateral n-type Ge_1−x_Sn_x_ and Si_1−x−y_Ge_y_Sn_x_ on SOI transistors were fabricated using a top-down gate-last JNT approach. The fabrication process requires further improvements, but back-gated single-nanowire JNTs have large *I*_*on*_/*I*_*off*_-ratios of about 1 × 10^8^. The top-gated JNTs have smaller *I*_*on*_/*I*_*off*_-ratios of about 1 × 10^3^ (Ge_1−x_Sn_x_) and 1 × 10^5^ (Si_1−x−y_Ge_y_Sn_x_) respectively. The combined application of *V*_*BG*_ and *V*_*TG*_ potentials simulates a kind of gate-all-around JNT architecture, which allows one to drive the JNT to an operating point by using a constant *V*_*BG*_ and then modulating the current by the *V*_*TG*_ sweep. By this measure, the large *I*_*on*_/*I*_*off*_-ratios from the back-gated JNT can be achieved with a much steeper JNT switching by the top gate.

## Supplementary Information

Below is the link to the electronic supplementary material.

Supplementary Material 1

## Data Availability

Data is provided within the manuscript or supplementary information files.
