# Supplementary material for: Fabrication and characterization of n-type Ge1−xSnx- and Si1–x–yGeySnx-on-SOI junctionless transistors
Source: Sci Rep. 2025 Dec 26;15:44659. doi: 10.1038/s41598-025-31272-y (PMC12749494; doi:10.1038/s41598-025-31272-y)
Supplement: Supplementary file 1 — Supplementary Material 1 [file 41598_2025_31272_MOESM1_ESM.pdf]

n-type $Ge_{1-x}Sn_x$- and $Si_{1-x-y}Ge_ySn_x$-on-SOI junctionless transistors

Oliver Steuer[1,4], Sayantan Ghosh[1], Daniel Schwarz[2], Michael Oehme[2], Sebastian Lehmann[3], René Hübner[1], Ciarán Fowley[1], Artur Erbe[1], Shengqiang Zhou[1], Manfred Helm[1,6], Gianaurelio Cuniberti[4], Slawomir Prucnal[1], and Yordan M. Georgiev[1,5]

[1] Institute of Ion Beam Physics and Materials Research, Helmholtz-Zentrum Dresden-Rossendorf, Bautzner Landstraße 400, 01328 Dresden, Germany

[2] University of Stuttgart, Institute of Semiconductor Engineering, Pfaffenwaldring 47, 70569 Stuttgart, Germany

[3] Institute for Metallic Materials, Leibniz Institute of Solid State and Materials Science, 01069 Dresden, Germany

[4] Institute of Materials Science and Max Bergmann Center, Technische Universität Dresden, 01069 Dresden, Germany

[5] Institute of Electronics, Bulgarian Academy of Sciences, 72, Tsarigradsko Chausse Blvd.,1784 Sofia, Bulgaria

[6] Center for Advancing Electronics Dresden, Technische Universität Dresden, 01062 Dresden, Germany

E-mail: o.steuer@hzdr.de, s.prucnal@hzdr.de

Supplementary materials

A) Operation principle of n-type JLFETs

The operating principle of n-type JLFET is based on the modulation of carriers by applying an electrical field through the gates. The source, channel, and drain regions are highly n-type doped, which increases the semiconductor conductivity and improves the source/drain contact properties [1, 2].

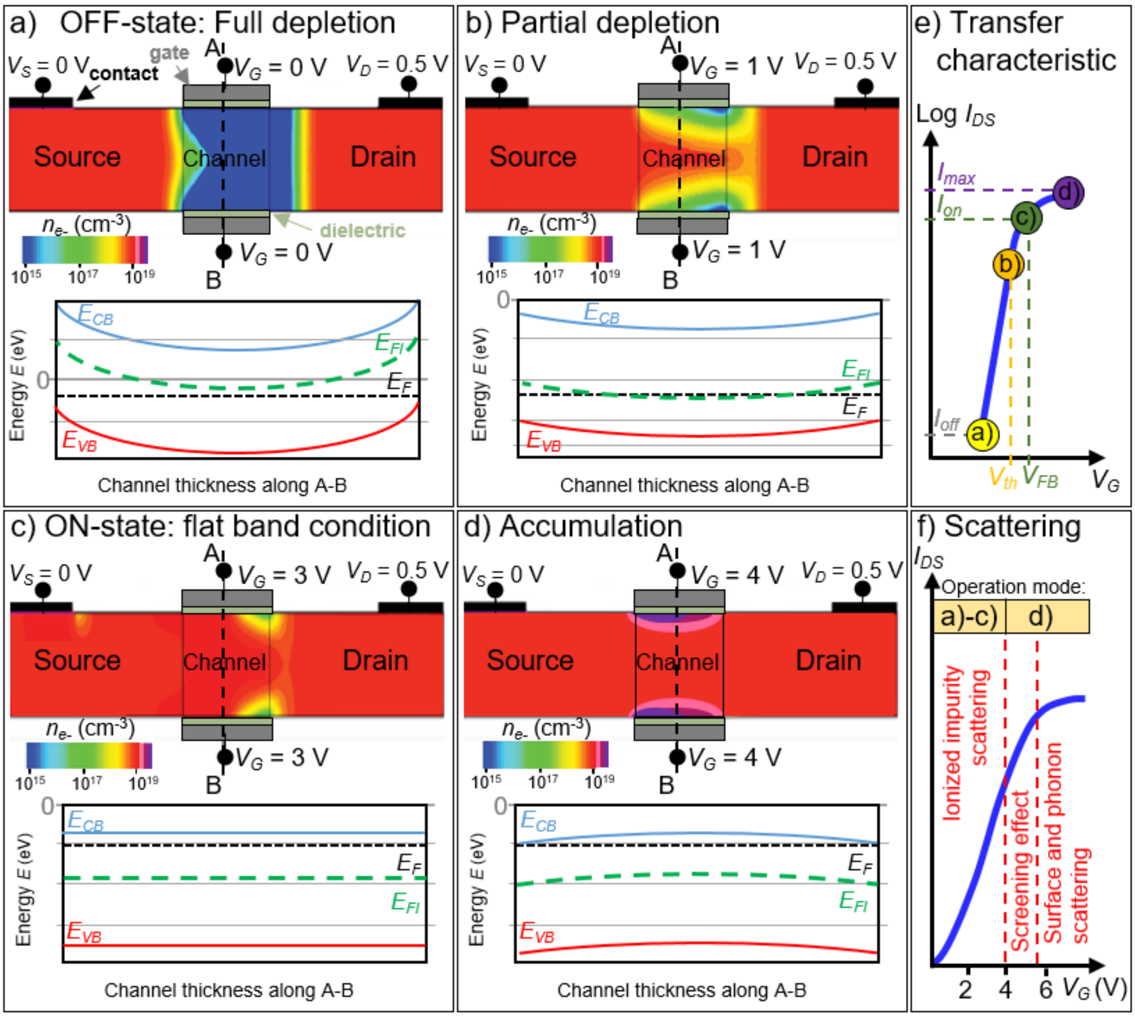

Fig. A 1: Schematic electron concentration contour plots and energy band diagrams of selected n-type double-gate JLFET operation modes (a) - d)) in analogy to ref. [2]. The color code within the device is related to the carrier concentration n_{e-} (see legend below each device schematic). The band diagrams are taken across the channel thickness (along the A-B line), and E_{CB} is the conduction band energy (blue solid line), E_F is the Fermi energy (dashed black line), E_{FI} is the intrinsic Fermi energy (green dashed line), and E_{VB} is the valence band energy (red solid line). The contour plot shows the electron concentration n_{e-} for different applied gate voltages V_G in the full depletion a), partial depletion b), flat-band condition c), and accumulation d) mode. Schematic transfer characteristic of an n-type JNT with a logarithmic depiction of the drain-source current I_{DS} in dependence on V_G at a constant source V_S - drain V_D potential e). I_{off} is the off-current, V_{th} is the threshold voltage related to the partial depletion mode of b), I_{on} is the on-current at V_{FB} (the flat-band voltage) related to the flat-band condition of c), and I_{max} is the enhanced on-current in the accumulation mode of d). Primary scattering mechanisms depending on the different operation modes for a linearly scaled transfer characteristic f). Due to different scattering mechanisms, the accumulation region is divided into weak (small V_G) and strong accumulation (large V_G).

Adding a thin dielectric and a gate material forms a metal-oxide-semiconductor (MOS) capacitor structure at the channel region [3]. If the work function of the gate is larger than the n-type semiconductor, the channel beneath the gate can be depleted of e^-, as shown in Fig. A 1 a) for the full volume depletion in the JLFET channel. To ensure a full volume depletion, it is required to adapt the channel shape, gate work function, dopant concentration, and dielectric thickness to each other. The functionality of a JNT and a double-gate JLFET is qualitatively the same. However, the NW channel shape allows the control of the channel by the surrounding gate to be more effective due to the additional electrical field from the NW side surfaces. The carrier depletion increases the resistance and turns the n-type normally-OFF JLFET off. Applying a certain positive gate voltage V_G moves the Fermi energy E_F above the intrinsic Fermi energy E_{FI} in the band diagram of Fig. A 1 b). This reduces the depletion layer width and uncovers an undepleted conducting cross section with a high carrier concentration in the channel center, as shown in Fig. A 1 b) [4]. The required gate potential to achieve a partial depletion condition is defined as the threshold voltage V_{th} of a JLFET [2]. At this stage, the e^- move from source to drain within the channel center following bulk conduction. Hence, the drain-source current I_{DS} increases significantly in Fig. A 1 e). Further increase of V_G uncovers a larger conducting cross section until the entire channel cross section is conducting at the so-called flat-band voltage V_{FB}, which is associated with the JLFET on-current I_{on} (see Fig. A 1 e)) [4]. The related energy band diagram of the flat-band condition in Fig. A 1 c) depicts the bands as straight lines, and E_F is entirely above E_{FI}. Owing to the device architecture, the electrical fields inside the gate electrode are aligned perpendicular to the channel thickness for the double-gate structure or NW diameter for the gate-all-around case. Hence, the minimum field in the flat-band condition is located in the channel/NW center. The absence of the electric field and surface-related defects in the center increase the mobility and favor bulk conduction in the ON-state [3 - 6]. However, experimental and simulation results revealed similar carrier mobilities of JLFETs in bulk conduction mode compared to MOSFETs in surface conduction mode, since the high dopant concentration in the JLFET channel leads to enhanced Coulomb scattering [2, 6, 7]. In general, Coulomb scattering is the most dominant scattering mechanism of the JLFET operation modes discussed so far, as highlighted in Fig. A 1 f). Further increasing V_G causes an accumulation of e^- at the dielectric-semiconductor interface, as shown in the contour plot in Fig. A 1 d), and bends the energy bands downward close to the semiconductor surface. In the case of a weak accumulation, a "screening effect" of the ionized dopants reduces impurity scattering, which increases I_{DS} in Fig. A 1 f). The screening effect is based on the opposite polarities of the carriers and the ionized dopants [6]. The negative e^- are attracted by the positively ionized dopants and are assembled around the dopants. At the same time, the charge field of the attracted e^- shields the conducting e^- from being scattered by impurities, which increases the channel mobility in the accumulation layer.

This effect can be expanded up to a volume accumulation of the channel region. However, for small NW dimensions, scattering at the NW surface and phonon scattering become present in the strong accumulation mode, which limits I_{DS}, as shown in Fig. A 1 f). In dependence on the JLFET design, sophisticated models based on solving Poisson´s equation were developed for different operation regimes and are summarized in ref. [8].

B) Extraction of the JNT figures of merits

Flat-band voltage V_{FB} and on-current I_{on}

The flat-band voltage V_{FB} is the gate voltage V_G at which the JNT channel is entirely open in the flat-band condition. This means the JNT is turned on, and the I_{on} can flow between the drain and the source. V_{FB} is located at the maximum of transconductance g_m, which can be calculated using Eq. B 1.

$$g_m = \frac{dI_{DS}}{dV_G} \qquad \text{Eq. B 1}$$

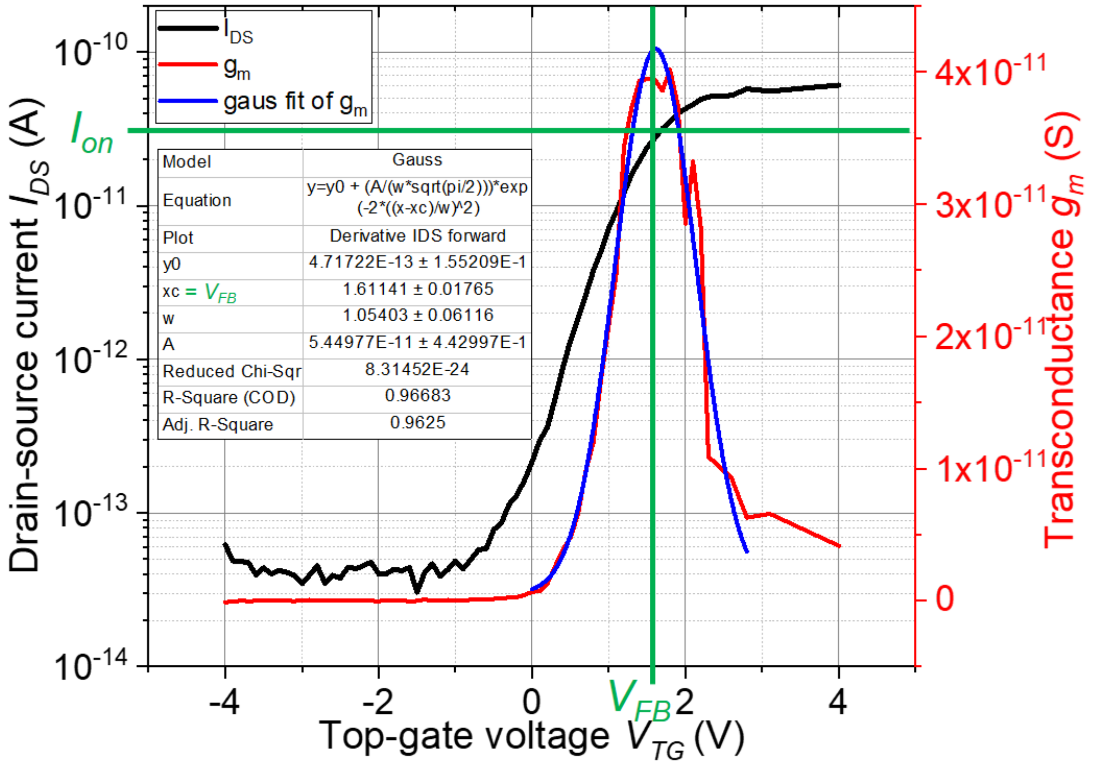

Fig. B 1: Extraction of V_{FB} and I_{on} using the transconductance g_m and a Gauss peak fit of g_m.

I_{off}, I_{max}, I_{on}/I_{off}- and I_{max}/I_{off}-ratios

The off-current I_{off} is the leakage current between the drain and the source in the JNT depletion mode. This means that the channel is entirely depleted of carriers, which prevents a sufficient current flow. On the other hand, the maximum drain-source current I_{max} is the I_{DS} in the accumulation mode of the JNT. The I_{off} and I_{max} parameters can be obtained directly from the transfer characteristic. In this work, the transfer characteristics were determined with a double sweep from 0 V to +40 V to -40 V to +40 V to -40 V to 0 V for the back-gating case or from 0 V to +4 V to -4 V to +4 V to -4 V to 0 V for the top-gating case. Then, I_{off} was defined as the measured minimum I_{DS} and I_{max} as the maximum I_{DS} of the forward sweep between -40 V and +40 V (back-gating) or -4 V and +4 V (top-gating). The I_{max}/I_{off}-ratio as well as the I_{on}/I_{off}-ratio,

were finally calculated by the transistor figures of merits, which describe the ratio between I_{max} and I_{off} or I_{on} and I_{off}

Subthreshold swing *SS* calculation

The subthreshold swing *SS* is defined as the amount of voltage required to change the subthreshold current by one order of magnitude (decade). This can be approximated from the transfer characteristic with the drain-source current I_{DS} in a logarithmical scale and the gate voltage V_G in a linear scale with the help of Eq. B 2. Since the slope in the subthreshold region is not everywhere exactly the same, it is common to select the steepest subthreshold slope (SS^{-1}) by using the inflection point of the logarithmic I_{DS}-V_G plot. In this work, the inflection point was placed in the middle of I_{DS1} and I_{DS2}. Furthermore, a distance of approximately one decade is selected between I_{DS1} and I_{DS2}.

$$SS = \frac{\Delta V_G}{\log\left(\frac{I_{DS1}}{I_{DS2}}\right)} \quad \text{Eq. B 2}$$

C) Discussion about the thin $Ge_{1-x}Sn_x$ and $Si_{1-x-y}Ge_ySn_x$ layers

The original material consisted of a 20 nm-thick $Si_{0.14}Ge_{0.80}Sn_{0.06}$ or $Ge_{0.94}Sn_{0.06}$ top layer doped with Sb (5×10^{19} cm^{-3}) grown on a silicon-on-insulator substrate with a 20 nm-thick slightly p-type doped top Si layer. The large lattice parameter mismatch between the $Ge_{1-x}Sn_x$ or $Si_{1-x-y}Ge_ySn_x$ alloy and the SOI resulted in defect-rich Sn-containing layers (see ref. [9]). Due to the etching processes during the device fabrication, the original 20 nm-thick $Ge_{0.94}Sn_{0.06}$ or $Si_{0.14}Ge_{0.80}Sn_{0.06}$ layers became rough and significantly thinner, as exemplarily shown in Fig. C 1 a) - b) at the microscale and Fig. C 1 c) - e) at the nanoscale. The observed roughness and reduction in the layer thickness is most likely related to an enhanced etching at defect sites formed during the $Ge_{1-x}Sn_x$ or $Si_{1-x-y}Ge_ySn_x$ growth. Nevertheless, the presence of a thin Ge-rich layer from the Ge-based alloys is clearly visible in Fig. C 1 b). On the other hand, the Sn signal from these Sn-containing layer is not observable in Fig. C 1 b). The investigation of the nanowire structure at higher magnification, as presented in Fig. C 1 c)-e), reveals that Sn is barely detectable in these very thin layers. Only in regions where the thickness exceeds approximately 4 nm, the presence of Sn is confirmed. However, it must be noted that the low Sn concentration and the thin layer thickness are challenges for a precise analysis of the EXDS data.

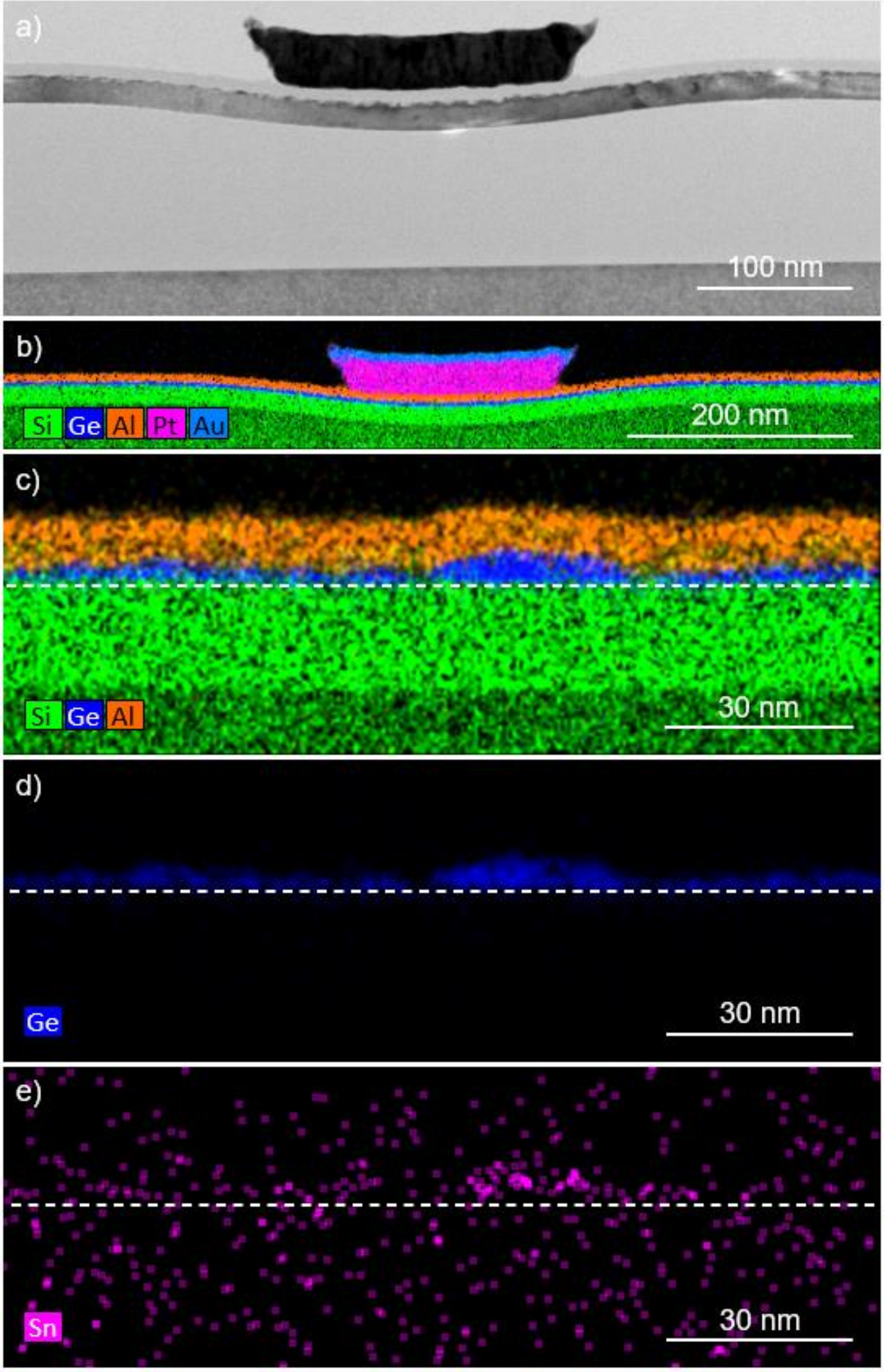

Fig. C 1: Cross-sectional TEM-based analysis along the $Si_{1-x-y}Ge_ySn_x$ nanowire (NW) of the $Si_{1-x-y}Ge_ySn_x$ JNTs (a)-e)). The TEM image a) with the Pt/Au top gate in the center serves as an overview for the EDXS-based element distribution analysis b) showing a superposition of Si in green, Ge in blue, Al in orange, Pt in magenta, and Au in light blue. Magnified EDXS-based element distribution analysis showing a superposition of Si in green, Ge in blue, and Al in orange c) and single element maps for Ge d) and Sn e). The white dashed line was inserted in c)-e) to emphasize the position of the interface between the top silicon of the SOI wafer and the $Si_{1-x-y}Ge_ySn_x$.

D) Benchmark and future prospects

In recent years, some $Ge_{1-x}Sn_x$ transistors [10-21] have been published. However, only a few n-type $Ge_{1-x}Sn_x$ transistors [11, 18, 22-25] have been presented among these results because of difficulties like the unintentional p-type background doping, in-plane compressive strain, and Fermi level pinning. To the best of our knowledge, no ternary $Si_{1-x-y}Ge_ySn_x$ transistors have been reported yet. Even though it is difficult to compare the different device concepts directly because of different functionalities, doping levels, alloy composition, device dimensions, etc., the I_{on}/I_{off}-ratio versus the *SS* in Fig. D 1 gives an overview of the n-type transistor performances achieved so far. Details about the transistors are summarized in Table D 1. Regarding the drain-source I_{on}/I_{off}-ratios, the presented top-gated $Ge_{1-x}Sn_x$ and $Si_{1-x-y}Ge_ySn_x$ JNTs are among the better performing devices. Furthermore, the application of an additional back-gate voltage while modulating the $Ge_{1-x}Sn_x$ JNTs current with the top-gate allowed to boost the I_{on}/I_{off}-ratio from 1×10^3 to record-values of up to 1×10^8. Such high I_{on}/I_{off}-ratios can be achieved since the $Ge_{1-x}Sn_x$ JNTs can be turned off entirely, which is essential for low standby power technologies. The achievable off-currents as low as <10 fA and large I_{on}/I_{off}-ratios benefit from the JNT device concept on an insulating substrate. On the other hand, significantly faster $Ge_{1-x}Sn_x$ transistors with an almost ideal *SS* of 76 mV $dec.^{-1}$ have recently been achieved for vertical gate all around (GAA) $Ge_{0.95}Sn_{0.05}$ FETs [11]. Such small *SS* can be achieved due to high material quality, low interface defect densities, and good gate control, which proves the existence of suitable process windows for many potential applications. Here, the vertical device concept seems advantageous over the lateral configuration, since the fabrication approach allows the growth of thick Ge-buffer layers, which helps to reduce the defect concentration of the active $Ge_{1-x}Sn_x$ layer. A similar growth approach was used for GeSnOI in ref. [22] before transferring the GeSn layer to the insulating substrate. As shown for a Si JNT with a NW diameter of about 10 nm, the device concept of JNTs is generally capable of reaching small *SS,* such as 70 mV $dec.^{-1}$ [26].

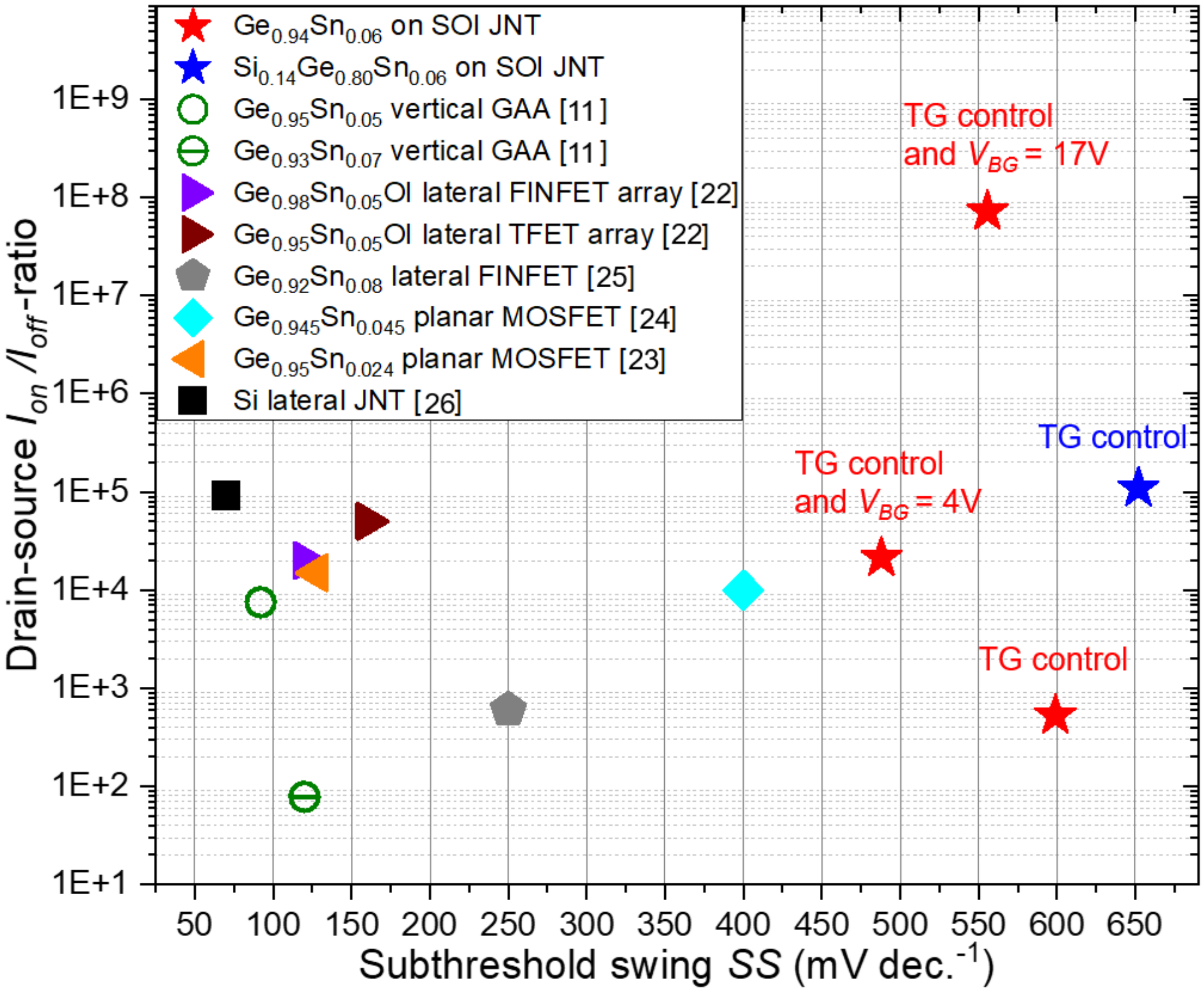

Fig. D 1: Benchmark of the fabricated n-type $Ge_{1-x}Sn_x$ and $Si_{1-x-y}Ge_ySn_x$ on SOI JNTs (symbol star) with n-type transistors in different device configurations measured at a constant supply voltage V_{DS} = 0.5 V for $Ge_{1-x}Sn_x$ and $Si_{1-x-y}Ge_ySn_x$ [11, 22-26]. Details about the compared transistors are summarized in Table D 1. The supply voltage for the Si lateral JNT was V_{DS} = 0.9 V.

It is assumed that the device performance of the reported $Ge_{1-x}Sn_x$ and $Si_{1-x-y}Ge_ySn_x$ can be further improved by the following measures: i) aiming for thin and short NW geometries in the range of tens of nanometers in width, thickness and length as well as GAA gate structures with W_{TG} entirely covering L_{NW}, ii) improving the contact quality by increasing the source/drain doping level or applying contact formation annealing, iii) improving the $Ge_{1-x}Sn_x$ and $Si_{1-x-y}Ge_ySn_x$ layer crystal structures by reducing the lattice parameter mismatch between the alloy and the substrate, inserting a Ge buffer layer, applying non-equilibrium post growth thermal treatments [9] or directly using $Ge_{1-x}Sn_xOI$ [27] or $Si_{1-x-y}Ge_ySn_xOI$.

Table D 2: Summary of the n-type $Ge_{1-x}Sn_x$ and $Si_{1-x-y}Ge_ySn_x$ device parameters compared in Fig. D 1.

Year	Reference	SS (mV dec.$^{-1}$)	I_{on}/I_{off}-ratio	As-grown channel material	Device structure
2025	This work	599	5.3 x 10^2	$Ge_{0.94}Sn_{0.06}$ on SOI	$Ge_{1-x}Sn_x$ on SOI JNT with a L_{NW} = 500 nm long, W_{NW} = 90 nm wide NW, and a W_{TG} = 140 nm wide top-gate. The measurement was performed with V_{DS} = 0.5 V by sweeping the top-gate V_{TG} = ±4 V with V_{BG} = 0 V.
2025	This work	488	2.2x 10^4	$Ge_{0.94}Sn_{0.06}$ on SOI	$Ge_{1-x}Sn_x$ on SOI JNT with a L_{NW} = 500 nm long, W_{NW} = 90 nm wide NW, and a W_{TG} = 140 nm wide top-gate. The measurement was performed with V_{DS} = 0.5 V by sweeping the top-gate V_{TG} = ±4 V and under a constant back-gate potential of V_{BG} = 4 V.
2025	This work	555	7.2 x 10^7	$Ge_{0.94}Sn_{0.06}$ on SOI	$Ge_{1-x}Sn_x$ on SOI JNT with a L_{NW} = 500 nm long, W_{NW} = 90 nm wide NW, and a W_{TG} = 140 nm wide top-gate. The measurement was performed with V_{DS} = 0.5 V by sweeping the top-gate V_{TG} = ±4 V and under a constant back-gate potential of V_{BG} = 17 V.
2025	This work	625	1.1 x 10^5	$Si_{0.14}Ge_{0.80}Sn_{0.06}$ on SOI	$Si_{0.14}Ge_{0.80}Sn_{0.06}$ on SOI JNT with a NW length of L_{NW} = 500 nm, width of W_{NW} = 219 nm, and top-gate width of W_{TG} = 219 nm. The measurement was performed with V_{DS} = 0.5 V by sweeping the top-gate
2023	[22]	92	7.5 x 10^3	$Ge_{0.95}Sn_{0.05}$	Vertical GAA n-FET with a NW diameter of 25 nm and gate length of 100 nm. The structure consists of a $GeSnO_x$/1 nm Al_2O_3/ 5 nm HfO_2 dielectric, and a 40 nm TiN gate. The measurement was performed with V_{DS} = 0.5 V.
2023	[22]	120	7.5 x 10^1	$Ge_{0.922}Sn_{0.078}$	Vertical GAA n-FET with a NW diameter of 50nm and gate length of 70 nm; The dielectric is $GeSnO_x$/1 nm Al_2O_3/ 5 nm HfO_2, and the gate material is 40 nm TiN. The measurement was performed with V_{DS} = 0.5V.
2019	[22]	120	2 x 10^4	$Ge_{0.95}Sn_{0.05}$	Lateral $Ge_{0.95}Sn_{0.05}$OI n-FinFET array (5 fins) with a fin width of 20 nm, fin height of 50 nm, a gate dielectric of 4 nm HfO_2, and a Mo/W gate. The measurement was performed with V_{DS} = 0.5 V.
		160	5 x 10^4	$Ge_{0.95}Sn_{0.05}$	Lateral $Ge_{0.95}Sn_{0.05}$OI n-TFET array (5 fins) with a fin width of 20 nm, fin height of 50 nm, a gate dielectric of 4 nm HfO_2, and a Mo/W gate. The measurement was performed with V_{DS} = 0.5 V.
2017	[25]	250	6 x 10^2	$Ge_{0.92}Sn_{0.08}$ on Ge-buffered SOI	$Ge_{0.92}Sn_{0.08}$ inversion mode n-FinFETs with a fin width between 40 nm and 100 nm and $Ge_{0.92}Sn_{0.08}$ height of 50 nm. The gate dielectric is $GeSnO_x$ + Al_2O_3, and a TiN top-gate was used. The measurement was performed with V_{DS} = 0.5 V.
2015	[24]	400	1 x 10^4	$Ge_{0.955}Sn_{0.045}$ on Ge	Planar $Ge_{0.955}Sn_{0.045}$ n-MOSFETs with a channel width of 80 µm and length of 20 µm. The dielectric is $GeSnO_x$ / 20 nm Al_2O_3, and TaN is used as the top-gate. The measurement was performed with V_{DS} = 0.5 V.
2012	[23]	128	1.5 x 10^4	$Ge_{0.976}Sn_{0.024}$ on Ge	Planar $Ge_{0.976}Sn_{0.024}$ n-MOSFETs with a gate length of 6.5µm. The dielectric is $GeSnO_2$ and 6 nm Al_2O_3, and TaN was used as gate metal. The measurement was performed with V_{DS} = 0.5 V.

2012	[26]	70	9.1 x 10^4	Si	Lateral SOI JNT with an n-type doping concentration of P with n_{e-} = 4 x 10^{19} cm^{-3}. The vertical NW height is d_{NW} = 9 nm, and the gate length is W_{TG} = 13 nm. A 2.3 nm HfSiON (EOT ≈ 1.2 nm) dielectric and a top-gate with 5 nm TiN and 50 nm polysilicon were used. This JNT was measured at V_{DS} = 0.9V.

References

[1] C.-W. Lee, A. Afzalian, N.D. Akhavan, R. Yan, I. Ferain, J.-P. Colinge, Junctionless multigate field-effect transistor, Appl Phys Lett 94 (2009) 053511. https://doi.org/10.1063/1.3079411

[2] M.J.K. Shubham Sahay, Junctionless Field-Effect Transistors, IEEE Press Series on Microelectronic Systems, Piscataway, 2019, https://books.google.de/books?hl=de&lr=&id=0feEDwAAQBAJ&oi=fnd&pg=PR11&dq=Junctionless+Field-Effect+Transistors&ots=DIRdcBLpiR&sig=BG0VYaiWxZdnwUknlqgoKPz5pfY&redir_esc=y#v=onepage&q=Junctionless%20Field-Effect%20Transistors&f=falseISBN 978-1-119-52353-6.

[3] C.-W. Lee, I. Ferain, A. Afzalian, R. Yan, N.D. Akhavan, P. Razavi, J.-P. Colinge, Performance estimation of junctionless multigate transistors, Solid-State Electronics 54 (2010) 97-103. https://doi.org/10.1016/j.sse.2009.12.003

[4] J.P. Colinge, C.W. Lee, A. Afzalian, N.D. Akhavan, R. Yan, I. Ferain, P. Razavi, B. O'Neill, A. Blake, M. White, A.M. Kelleher, B. McCarthy, R. Murphy, Nanowire transistors without junctions, Nat Nanotechnol 5 (2010) 225-229. https://doi.org/10.1038/nnano.2010.15

[5] R. Rios, A. Cappellani, M. Armstrong, A. Budrevich, H. Gomez, R. Pai, N. Rahhal-orabi, K. Kuhn, Comparison of Junctionless and Conventional Trigate Transistors With Lg Down to 26 nm, IEEE Electron Device Letters 32 (2011) 1170-1172. https://doi.org/10.1109/LED.2011.2158978

[6] K.-I. Goto, T.-H. Yu, J. Wu, C.H. Diaz, J.P. Colinge, Mobility and screening effect in heavily doped accumulation-mode metal-oxide-semiconductor field-effect transistors, Appl Phys Lett 101 (2012) 073503. https://doi.org/10.1063/1.4745604

[7] N. Kadotani, T. Ohashi, T. Takahashi, S. Oda, K. Uchida, Experimental Study on Electron Mobility in Accumulation-Mode Silicon-on-Insulator Metal–Oxide–Semiconductor Field-Effect Transistors, Japanese Journal of Applied Physics 50 (2011) 094101. https://doi.org/10.1143/JJAP.50.094101

[8] J.-P. Colinge, Emerging Devices for Low-Power and High-Performance Nanosystems: Physics, Novel Functions, and Data Processing, Pan Stanford Publishing Pte. Ltd.2019, 978-981-4800-11-2.

[9] O. Steuer, D. Schwarz, M. Oehme, F. Bärwolf, Y. Cheng, F. Ganss, R. Hübner, R. Heller, S. Zhou, M. Helm, G. Cuniberti, Y.M. Georgiev, S. Prucnal, Structural changes in Ge1−xSnx and Si1−x−yGeySnx thin films on SOI substrates treated by pulse laser annealing, Journal of Applied Physics 136 (2024). https://doi.org/10.1063/5.0218703

[10] D. Lei, K.H. Lee, Y.-C. Huang, W. Wang, S. Masudy-Panah, S. Yadav, A. Kumar, Y. Dong, Y. Kang, S. Xu, Y. Wu, C.S. Tan, X. Gong, Y.-C. Yeo, Germanium-Tin (GeSn) P-Channel Fin Field-Effect Transistor Fabricated on a Novel GeSn-on-Insulator Substrate, IEEE Transactions on Electron Devices 65 (2018) 3754-3761. https://doi.org/10.1109/TED.2018.2856738

[11] M. Liu, Y. Junk, Y. Han, D. Yang, J.H. Bae, M. Frauenrath, J.-M. Hartmann, Z. Ikonic, F. Bärwolf, A. Mai, D. Grützmacher, J. Knoch, D. Buca, Q.-T. Zhao, Vertical GeSn nanowire MOSFETs for CMOS beyond silicon, Communications Engineering 2 (2023) 7. https://doi.org/10.1038/s44172-023-00059-2

[12] L. Zhang, H. Hong, C. Yu, C. Li, S. Chen, W. Huang, J. Wang, H. Wang, Poly-GeSn Junctionless Thin-Film Transistors on Insulators Fabricated at Low Temperatures via Pulsed Laser Annealing, physica status solidi (RRL) – Rapid Research Letters 13 (2019) 1900420. https://doi.org/10.1002/pssr.201900420
[13] C.-P. Chou, Y.-X. Lin, K.-Y. Hsieh, Y.-H. Wu, Poly-GeSn junctionless P-TFTs featuring a record high ION/IOFF ratio and hole mobility by defect engineering, Journal of Materials Chemistry C 7 (2019) 5201-5208. https://doi.org/10.1039/C8TC04972F
[14] C.-P. Chou, Y.-X. Lin, Y.-H. Wu, Implementing P-Channel Junctionless Thin-Film Transistor on Poly-Ge0.95Sn0.05 Film Formed by Amorphous GeSn Deposition and Annealing, IEEE Electron Device Letters 39 (2018) 1187-1190. https://doi.org/10.1109/LED.2018.2846882
[15] H. Oka, T. Amamoto, M. Koyama, Y. Imai, S. Kimura, T. Hosoi, T. Shimura, H. Watanabe, Fabrication of tensile-strained single-crystalline GeSn on transparent substrate by nucleation-controlled liquid-phase crystallization, Appl Phys Lett 110 (2017) 032104. https://doi.org/10.1063/1.4974473
[16] Y.-S. Huang, F.-L. Lu, Y.-J. Tsou, C.-E. Tsai, C.-Y. Lin, C.-H. Huang, C.W. Liu, 2017, First vertically stacked GeSn nanowire pGAAFETs with Ion = 1850μA/μm (Vov = Vds = −1V) on Si by GeSn/Ge CVD epitaxial growth and optimum selective etching, 2017 IEEE International Electron Devices Meeting (IEDM), 37,35,31-37,35,34. https://doi.org/10.1109/iedm.2017.8268512
[17] Y.-S. Huang, C.-H. Huang, F.-L. Lu, C.-Y. Lin, H.-Y. Ye, I.H. Wong, S.-R. Jan, H.-S. Lan, C.W. Liu, Y.-C. Huang, H. Chung, C.-P. Chang, S.S. Chu, S. Kuppurao, 2016, Record high mobility (428cm2/V-s) of CVD-grown Ge/strained Ge0.91Sn0.09/Ge quantum well p-MOSFETs, 2016 IEEE International Electron Devices Meeting (IEDM), 33,31,31-33,31,34. https://doi.org/10.1109/iedm.2016.7838531
[18] C. Schulte-Braucks, S. Glass, E. Hofmann, D. Stange, N. von den Driesch, J.M. Hartmann, Z. Ikonic, Q.T. Zhao, D. Buca, S. Mantl, Process modules for GeSn nanoelectronics with high Sn-contents, Solid-State Electronics 128 (2017) 54-59. https://doi.org/10.1016/j.sse.2016.10.024
[19] Y. Liu, J. Yan, H. Wang, B. Cheng, G. Han, Strained Germanium–Tin (GeSn) P-Channel Metal-Oxide-Semiconductor Field-Effect Transistors Featuring High Effective Hole Mobility, International Journal of Thermophysics 36 (2014) 980-986. https://doi.org/10.1007/s10765-014-1785-z
[20] X. Gong, G. Han, F. Bai, S. Su, P. Guo, Y. Yang, R. Cheng, D. Zhang, G. Zhang, C. Xue, B. Cheng, J. Pan, Z. Zhang, E.S. Tok, D. Antoniadis, Y.-C. Yeo, GermaniumTin (GeSn) p-Channel MOSFETs Fabricated on (100) and (111) Surface Orientations With 400°C Si2H6 Passivation, IEEE Electron Device Letters 34 (2013) 339-341. https://doi.org/10.1109/led.2012.2236880
[21] H. Genquan, S. Shaojian, Z. Chunlei, Z. Qian, Y. Yue, W. Lanxiang, G. Pengfei, W. Wang, W. Choun Pei, S. Ze Xiang, B. Cheng, Y.-C. Yeo, 2011, High-mobility germanium-tin (GeSn) P-channel MOSFETs featuring metallic source/drain and sub-370 °C process modules, 2011 International Electron Devices Meeting, 16,17,11-16,17,13. https://doi.org/10.1109/iedm.2011.6131569
[22] K. Han, Y. Wu, Y.C. Huang, S. Xu, A. Kumar, E. Kong, Y. Kang, J. Zhang, C. Wang, H. Xu, C. Sun, X. Gong, 2019, First Demonstration of Complementary FinFETs and Tunneling FinFETs Co-Integrated on a 200 mm GeSnOI Substrate: A Pathway towards Future Hybrid Nano-electronics Systems, 2019 Symposium on VLSI Technology, T182-T183. https://doi.org/10.23919/vlsit.2019.8776539
[23] G. Han, S. Su, L. Wang, W. Wang, X. Gong, Y. Yang, Ivana, P. Guo, C. Guo, G. Zhang, J. Pan, Z. Zhang, C. Xue, B. Cheng, Y.-C. Yeo, 2012, Strained germanium-tin (GeSn) N-channel MOSFETs featuring low temperature N+/P junction formation and

GeSnO2 interfacial layer, 2012 Symposium on VLSI Technology (VLSIT), 97-98. https://doi.org/10.1109/vlsit.2012.6242479
[24] Y.C. Fang, K.Y. Chen, C.H. Hsieh, C.C. Su, Y.H. Wu, N-MOSFETs Formed on Solid Phase Epitaxially Grown GeSn Film with Passivation by Oxygen Plasma Featuring High Mobility, ACS Appl Mater Interfaces 7 (2015) 26374-26380. https://doi.org/10.1021/acsami.5b08518
[25] Y. Chuang, H.-C. Huang, J.-Y. Li, 2017, GeSn N-FinFETs and NiGeSn contact formation by phosphorus implant, 2017 Silicon Nanoelectronics Workshop (SNW), 97-98. https://doi.org/10.23919/snw.2017.8242315
[26] S. Barraud, M. Berthome, R. Coquand, M. Casse, T. Ernst, M.P. Samson, P. Perreau, K.K. Bourdelle, O. Faynot, T. Poiroux, Scaling of Trigate Junctionless Nanowire MOSFET With Gate Length Down to 13 nm, IEEE Electron Device Letters 33 (2012) 1225-1227. https://doi.org/10.1109/LED.2012.2203091
[27] M. Wanitzek, M. Oehme, C. Spieth, D. Schwarz, L. Seidel, J. Schulze, 2022, GeSn-on-Si Avalanche Photodiodes for Short-Wave Infrared Detection, ESSCIRC 2022- IEEE 48th European Solid State Circuits Conference (ESSCIRC), 169-172. https://doi.org/10.1109/esscirc55480.2022.9911363
